# High Diversity of the Fungal Community Structure in Naturally-Occurring *Ophiocordyceps sinensis*

**DOI:** 10.1371/journal.pone.0015570

**Published:** 2010-12-15

**Authors:** Yongjie Zhang, Shu Zhang, Mu Wang, Fengyan Bai, Xingzhong Liu

**Affiliations:** 1 Key Laboratory of Systematic Mycology and Lichenology, Institute of Microbiology, Chinese Academy of Sciences, Beijing, China; 2 School of Life Sciences, Shanxi University, Taiyuan, China; 3 School of Plant Sciences and Technology, Agriculture and Animal Husbandry College of Tibet, Nyingchi, Tibet, China; University of Missouri-Kansas City, United States of America

## Abstract

**Background:**

*Ophiocordyceps sinensis* (syn. *Cordyceps sinensis*), which is a parasite of caterpillars and is endemic to alpine regions on the Tibetan Plateau, is one of the most valuable medicinal fungi in the world. “Natural *O. sinensis* specimens” harbor various other fungi. Several of these other fungi that have been isolated from natural *O. sinensis* specimens have similar chemical components and/or pharmaceutical effects as *O. sinensis*. Nevertheless, the mycobiota of natural *O. sinensis* specimens has not been investigated in detail.

**Methodology/Principal Findings:**

Based on the technique of PCR-single-strand conformation polymorphism (PCR-SSCP), the mycobiota of three different sections (stromata, sclerotia, and mycelial cortices) from natural *O. sinensis* specimens were investigated using both culture-dependent and -independent methods. For the culture-dependent method, 572 fungal strains were isolated, and 92 putative operational taxonomic units (OTUs) were identified from 226 sequenced strains with the threshold of 97%. For the culture-independent method, 490 fungal clones were identified from about 3000 clones of ITS fragments from the whole-community DNA; based on PCR-SSCP analyses, 266 of these clones were selected to be sequenced, and 118 putative OTUs were detected. The overwhelming majority of isolates/clones and OTUs were detected from mycelial cortices; only a few were detected from stromata and sclerotia. The most common OTUs detected with both methods belonged to Ascomycota; however, only 13 OTUs were detected simultaneously by both methods. Potential novel lineages were detected by each of the two methods.

**Conclusions/Significance:**

A great number of fungal species present in the mycobiota of naturally-occurring *O. sinensis* specimens were detected, and many of them may represent undescribed lineages. That only a few of the same OTUs were detected by both methods indicated that different methods should be used. This study increased our understanding about the fungal community structure of this valuable medicinal herb.

## Introduction

As a large, diverse, and economically important group of organisms, fungi impact humankind in a myriad of ways and are vital components of nearly all ecosystems and communities. The kingdom Fungi encompasses a broad range of taxa, morphologies, ecologies, and life-history strategies [1]. The total number of fungal species on Earth was estimated to be 1.5 million [2]. The number of fungal species that have been described, however, is only about 7% of this number [3].

The fungal community has been a focus of mycological research in recent years [4]. A large fraction of microorganisms in nature have so far not been cultivated in the laboratory [5], and therefore, culture-dependent as well as culture-independent methods should be used together to obtain a holistic view of fungal community structures [6]. In culture-dependent studies, fungal strains are isolated and then identified based on morphological characteristics and/or certain DNA sequences. In culture-independent studies, molecular techniques are used to construct libraries of certain DNA fragments, and clones are subsequently sequenced [6]. As a multi-copy and easily amplified region, the nrDNA internal transcribed spacer (ITS) region has become the standard locus for fungal identification, often even species demarcation, because of its high interspecific variability [7], [8]. The ITS regions are also valuable for studies of fungal communities [9] and DNA barcoding [10].

Fungal community studies often involve the identification of a large number of fungal species. Generally, many isolates (from a culture-dependent study) and clones (from a culture-independent study) that belong to identical lineages exist in the original sample pool of a fungal community. A simple and rapid analytical procedure should be applied before sequencing in order to identify operational taxonomic units (OTUs) quickly and economically.

Single-strand conformation polymorphism (SSCP) is an electrophoretic technique developed originally for the detection of mutations of DNA sequences [11]. SSCP can detect sequences that have subtle differences. This technique has been widely used in species identification and differentiation of human-, insect-, and plant-pathogenic fungi [12]–[15]; mycorrhizal fungi [16]; and yeasts [17]. This technique has also been used to study intraspecific genetic diversity of fungi [18]–[23]. As a community profiling technique, SSCP has also been applied to bacterial and fungal communities [24]–[26].

The entomopathogenic fungus *Ophiocordyceps sinensis* (Berk.) Sung, Sung, Hywel-Jones & Spatafora [≡*Cordyceps sinensis* (Berk.) Sacc.; anamorph: *Hirsutella sinensis* Liu, Guo, Yu & Zeng] [27], [28] parasitizes larvae (caterpillars) of moth species in the family Hepialidae. “Natural *O. sinensis* specimens”, the complex of the mummified caterpillar and the fungal stroma resulting from parasitism, are renowned in traditional Chinese medicine. Besides *O. sinensis*, 10 or more other fungal species have been isolated from natural *O. sinensis* specimens [29]–[31]. Some of these fungi can synthesize chemical components and/or have pharmaceutical value similar to *O. sinensis*, and fermented mycelial products have been developed and applied in clinical practice [32]. Nevertheless, the mycobiota of natural *O. sinensis* specimens has not been investigated in detail.

In this study, we investigated the fungal community structures of naturally-occurring *O. sinensis*. Many fungal cultures (for the culture-dependent study) and ITS clones (for the culture-independent study) were analyzed by PCR-SSCP before sequencing. A large number of OTUs including some unknown ones were identified. The potential significance of these OTUs is discussed.

## Materials and Methods

### Collection of natural *O. sinensis* specimens

Natural *O. sinensis* specimens were collected on the Tibetan Plateau in Southwestern China. The specimens (15 in total) were placed in a portable refrigerator, transported to our laboratory, and then stored at 4°C for no more than 2 weeks (for the culture-dependent investigation) or at −20°C until use (for the culture-independent investigation). As described in the next two sections, 11 specimens collected in Tibet Autonomous Region (AR) and Sichuan Province during 2004 and 2005 were used for the culture-dependent investigation, and four different specimens collected in Tibet AR and Qinghai Province in 2007 were used for the culture-independent investigation. These specimens were divided into three sections: stromata, sclerotia, and the complex of mycelial cortices and attached soil particles outside the sclerotia (abbreviated as mycelial cortices) (Fig. S1, Supporting Information).

### Culture-dependent community investigation

For the culture-dependent investigation, 11 natural *O. sinensis* specimens were used individually as samples. For mycelial cortices, fungi were isolated by the dilution-plate method. For stromata and sclerotia, these sections were first surface sterilized with 75% alcohol (30 s) and 0.1% mercury bichloride (3 min) and then cut into 1- to 2-mm-thick slices; these slices were ground in sterile water using a mortar and pestle, and homogenates were spread on potato dextrose agar (PDA) plates containing 0.1 g/L streptomycin and 0.05 g/L tetracycline. The plates were maintained in the dark at 20°C. Among the 572 fungal isolates obtained, 58 were from stromata, 60 were from sclerotia, and 454 were from mycelial cortices. Fungal isolates were first grouped according to their colony and morphological characteristics on PDA. Genomic DNA of isolates within each group was extracted with a simple and rapid “thermolysis” method as previously described [33], and the nrDNA ITS1 regions (between 18S and 5.8S rDNA) were amplified with primers ITS1 and ITS2 [34]. Amplicons were analyzed by PCR-SSCP (see *SSCP analysis* section), and the electrophoretic profiles were compared among isolates within each group. For strains showing different SSCP patterns, the full-length ITS regions (including ITS1, 5.8S, and ITS2) were amplified with primer pairs ITS1/ITS4 or ITS5/ITS4 [34], and sequences were determined by SinoGinoMax Co., Ltd. (China).

### Culture-independent community investigation

For the culture-independent investigation, four natural *O. sinensis* specimens were used individually as samples. Genomic DNA of the external mycelial cortices was extracted with the bead-beating method [35]. Stromata and sclerotia were first surface sterilized with the same procedures as above, and then genomic DNA was extracted with the traditional CTAB method [36]. The full-length nrDNA ITS regions were amplified from the genomic DNA with primers ITS1 and ITS4 [34], and the amplicons were then ligated into TA vector pMD18-T (TaKaRa, Japan) and cloned into *Escherichia coli* DH5α following the manufacturer's protocol. In total, 12 clone libraries were established for the four specimens. The nrDNA ITS1 regions were amplified from positive clones using primers ITS1 and ITS2 [34], and amplicons were analyzed by SSCP (see next section). A total of 200–250 positive clones for each library were analyzed by PCR-SSCP. The full-length ITS regions for representative clones showing different SSCP patterns were finally sequenced using the universal primer M13-47.

### SSCP analysis

SSCP screening of PCR products was conducted at 10°C using the DCodeTM Universal Mutation Detection System (Bio-Rad, Hercules, CA). PCR products (3 µl) were mixed with the same volume of denaturing buffer (95% formamide, 20 mM EDTA, 0.05% bromophenol blue, 0.05% xylene cyanol). The mixtures were heated at 95°C for 10 min and then immediately cooled on ice for 15 min. Denatured PCR products were then loaded into the slots of a polyacrylamide gel composed of 8% acrylamide-bisacrylamide (37.5:1), 1× TBE buffer, 5% glycerol, 0.1% ammonium persulfate, and 0.1% tetramethylethylenediamine (TEMED). After running for 5 min at 300 V, electrophoresis was continued for 8 h at 260 V. Finally, the gel was silver-stained [17]. Multiple SSCP analyses were performed when comparison of large numbers of isolates/clones was required.

### OTU definition and phylogenetic analysis

Sequences were aligned, and identical sequences were clustered using DNAMAN software (Version 6, Lynnon Corporation). An OTU was defined as a group of sequences with at least 97% pairwise similarity. One representative ITS sequence for each OTU was subsequently queried against GenBank using BLASTN [37], and whether one OTU was known or unknown was differentiated with the 97% sequence similarity criterion. All sequences for which the ITS1 and ITS2 regions matched unrelated sequences (i.e., different genera) from the database with an alignment score ≥200 were assumed to be chimeric sequences resulting from PCR recombination. Both chimeric sequences and sequences originating from plants, animals, and the fungus *O. sinensis* were excluded from subsequent analyses. An alignment of 5.8S rRNA gene sequences (between ITS1 and ITS2 regions, about 150 bp) was created, and neighbour-joining (NJ) and maximum parsimony (MP) phylogenetic analyses were carried out with 1000 replicates to produce bootstrap values using MEGA version 4 [38]. The most similar GenBank sequences were downloaded to be included in the phylogenetic tree construction, and the taxonomic status of each OTU was deduced with the assistance of annotations of these downloaded sequences. *Synchytrium macrosporum* (AY997095), *Synchytrium decipiens* (AY997094), and *Synchytrium puerariae* (EF053262) were used as the outgroup. The *Dictionary of the Fungi* [39] served as the source of taxonomic references for fungal species.

### Statistical analyses

Shannon-Weiner (*H'*) diversity indices were calculated with OTUs as a proxy for species using the program EstimateS 8.2 [40]. Species richness indices (*M*a) were calculated using the formula *M*a  =  (*S*-1)/ln *N* [41], in which *S* is the number of species (OTUs), and *N* is the total number of isolates or clones. The evenness (*J*) of fungal communities was represented by *J*  =  *H'*/*H' max* [42], where *H' max*  =  ln *S*.

The results of the culture-dependent and culture-independent investigations at the OTU level were compared by determining the similarity coefficient (*C*s) as follows: *C*s  = 2 *C*/(*A* + *B*) [43], where *A* and *B* are the number of OTUs detected from culture-dependent and culture-independent investigation, respectively, and *C* is the number of OTUs present in both investigations.

### Nucleotide sequence accession numbers

The ITS sequence of one representative isolate or clone for each fungal OTU was submitted to GenBank (http://www.ncbi.nlm.nih.gov/genbank/) and assigned accession numbers HM439513 to HM439604 and HQ445979 to HQ446096 for the sequences of cultivated strains and clones, respectively.

## Results

### Culture-dependent community investigation

**Figure 1:**
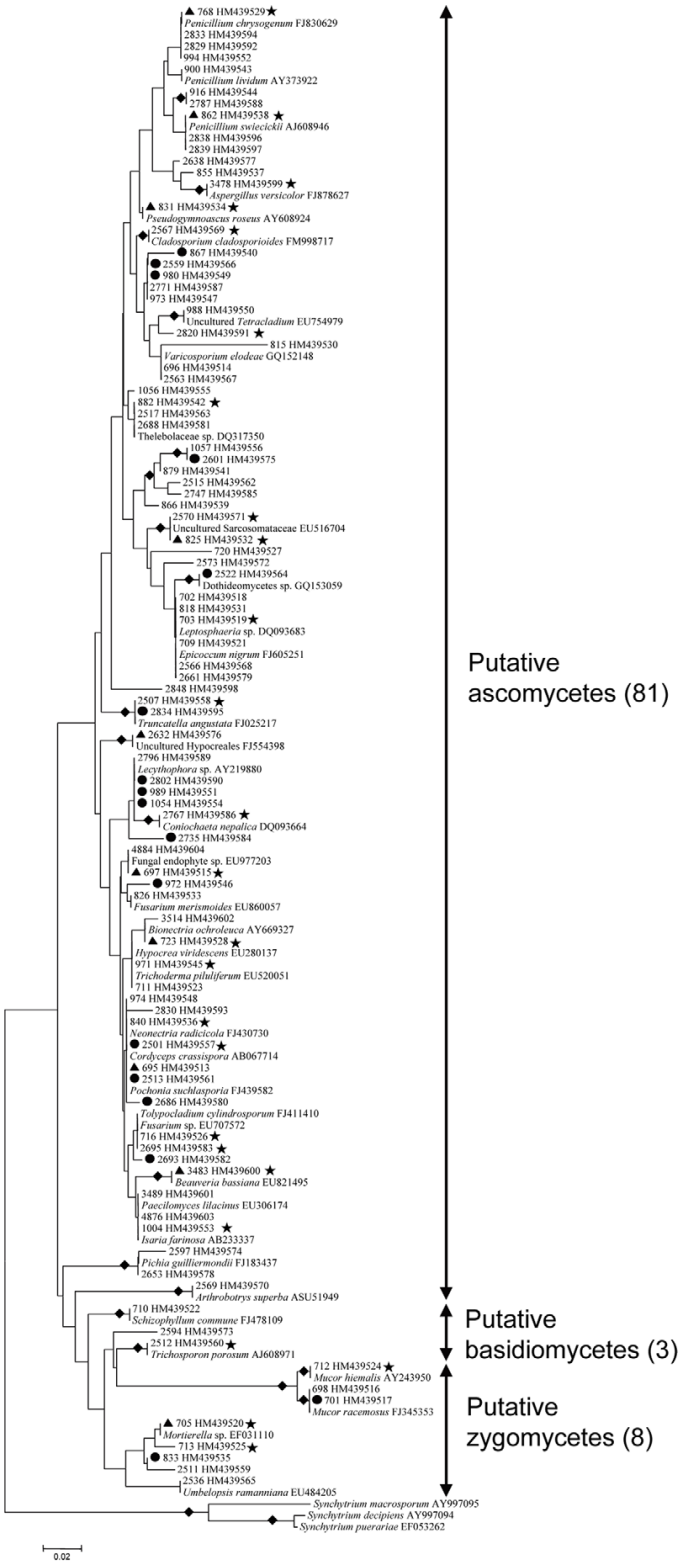
Molecular phylogeny of 5.8S rRNAs for OTUs detected in the culture-dependent investigation. Topologies of NJ and MP trees are almost identical, and only the NJ tree is shown here. The tree is based on a total of 133 sequences (92 OTUs detected in the culture-dependent investigation, 38 closest database matches of these OTUs and three outgroup sequences from GenBank) and 150 unambiguously aligned nucleotide positions. The 92 OTUs are indicated by storage numbers (three or four digits) of representative strains in our laboratory and GenBank accession numbers. Those 41 GenBank records are shown as they are annotated in the database, including their accession numbers. Solid diamonds indicate branches receiving more than 70% bootstrap values; solid circles represent potential new lineages; solid triangles represent OTUs that have a total abundance of more than 2%; solid stars indicate OTUs detected simultaneously from stromata (or sclerotia) and mycelial cortices of natural *O. sinensis* specimens.

**Figure 2:**
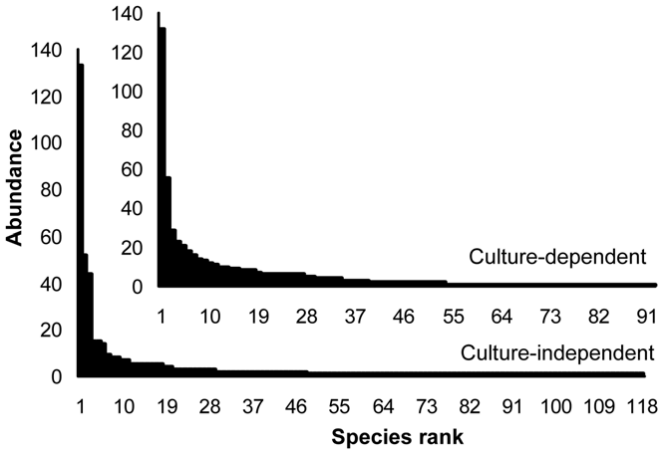
Rank-abundance plots for the culture-dependent and -independent investigations. Abundance of each OTU was indicated by the number of isolates in the culture-dependent investigation or the number of clones in the culture-independent investigation.

**Figure 3:**
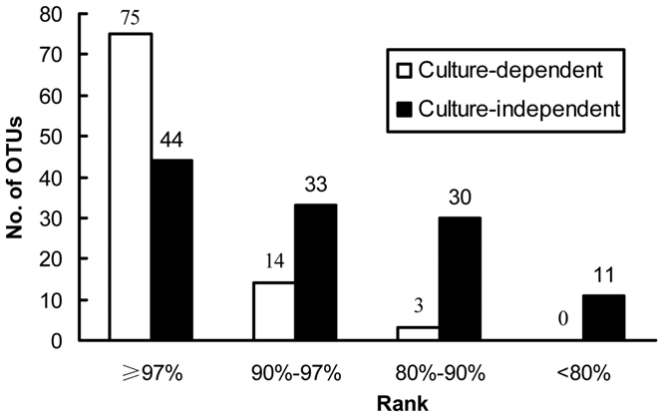
Distribution of OTUs of different similarity ranks with GenBank records. Those OTUs that have similarities greater than 97% with GenBank records were defined as known OTUs; those with less than 97% similarities were defined as unknown OTUs.

**Figure 4:**
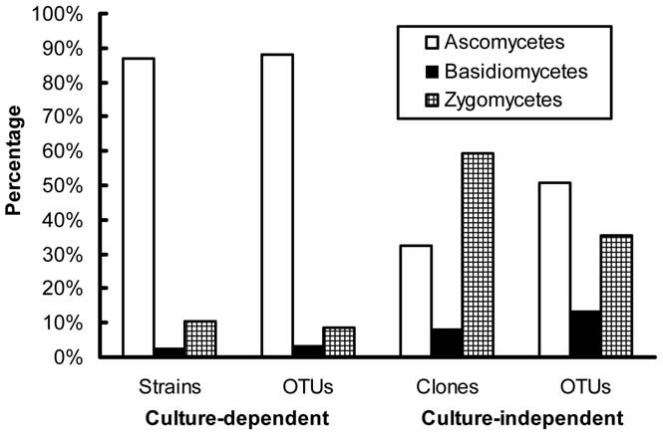
Percentage of strains/clones and OTUs of different phyla detected by the culture-dependent and -independent investigations.

In total, 572 fungal strains (58 from stromata, 60 from sclerotia, and 454 from mycelial cortices) were obtained by dilution plating. The nrDNA ITS1 regions of these isolates were analyzed by PCR-SSCP (Fig. S2A, Supporting Information). Full-length ITS sequences of 226 strains with different SSCP patterns were then amplified and sequenced. With the threshold of 97% for pairwise sequence similarities, 92 putative OTUs (27 from stromata, 19 from sclerotia, and 79 from mycelial cortices) were recognized; some OTUs were detected simultaneously from more than one section (Fig. 1). Thirty-nine OTUs (42% of all OTUs) occurred only once in the whole data set (Table S1), and the others occurred two to 132 times (0.3 to 23.1% of the data set) (Fig. 2). From BLASTN analysis, 75 OTUs were known (81.5%), and 17 were unknown (18.5%) (Fig. 1; Fig. 3). These known and unknown OTUs were affiliated with three phyla, namely, the Ascomycota (81 OTUs, 88.0%), the Basidiomycota (3 OTUs, 3.3%), and the Zygomycota (8 OTUs, 8.7%) (Fig. 1; Fig. 4).

### Culture-independent community investigation

**Figure 5:**
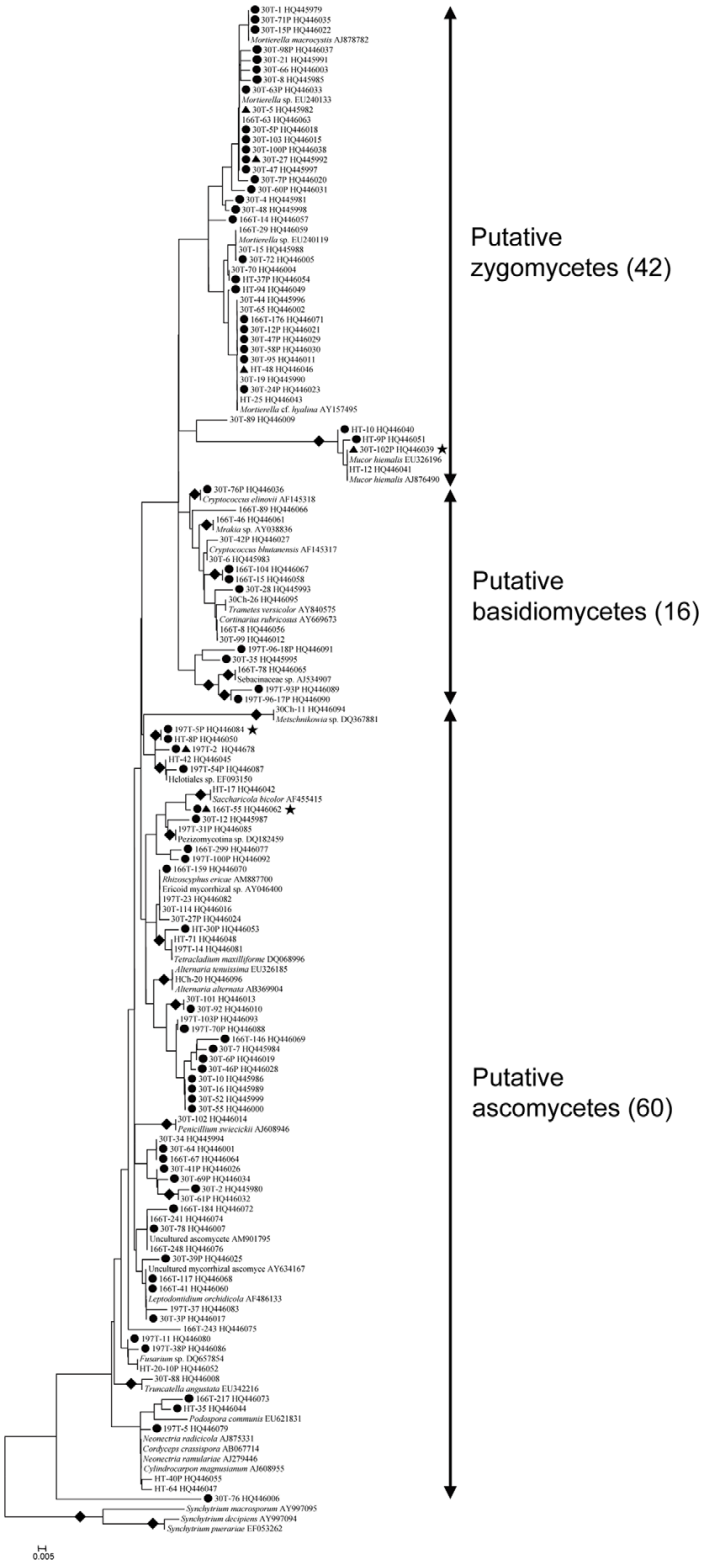
Molecular phylogeny of 5.8S rRNAs for OTUs detected in the culture-independent investigation. Topologies of NJ and MP trees are almost identical, and only the NJ tree is shown here. The tree is based on a total of 153 sequences (118 OTUs detected in the culture-independent investigation, 32 closest database matches of these OTUs and three outgroup sequences from GenBank) and 152 unambiguous nucleotide positions. OTUs are indicated by the name of representative clones and GenBank accession numbers. Those 35 GenBank records are shown as they are annotated in the database, including their accession numbers. Solid diamonds indicate branches receiving more than 70% bootstrap values; solid circles represent potential new lineages; solid triangles represent OTUs that have a total abundance of more than 2%; solid stars indicate OTUs detected simultaneously from stromata (or sclerotia) and mycelial cortices of natural *O. sinensis* specimens.

For the culture-independent investigation of the mycobiota of natural *O. sinensis* specimens, 12 DNA preparations (obtained from three kinds of tissue from each of four natural *O. sinensis* specimens) were used to generate ITS clone libraries. In total, 1028 clones from stromata, 1024 clones from sclerotia, and 891 clones from external mycelial cortices were analyzed by PCR-SSCP (Fig. S2B, S2C, Supporting Information). More than 98% of the clones from stromata and sclerotia represented *O. sinensis*; however, many different fungal clones were detected in external mycelial cortices. Clones of *O. sinensis* (2333 clones), plants (12), animals (5), and chimeras (13) were eliminated from the data set. For the remaining 490 fungal clones (2 from stromata, 6 from sclerotia, and 482 from mycelial cortices), 266 clones with different SSCP patterns were sequenced. In total, 118 unique OTUs (2 from stromata, 4 from sclerotia, and 115 from mycelial cortices) were identified based on ≥97% sequence similarity; some OTUs were detected simultaneously from more than one section (Fig. 5). Seventy of these OTUs (59.3%) were singletons occurring only once in the entire data set (Table S1), and the remaining 48 OTUs ranged in abundance from two to 133 clones (0.4 to 27.1% of the data set) (Fig. 2). By BLASTN analysis, 37.3% of these OTUs were known (44 OTUs), and 62.7% were unknown (74 OTUs) (Fig. 3; Fig. 5). Forty-one of these unknown OTUs have similarities lower than 90% with GenBank sequences (Fig. 3), and they may represent undocumented or novel fungal species. As for the 118 known and unknown OTUs, 60 were assigned to the Ascomycetes (50.8%), 16 were assigned to the Basidiomycetes (13.6%), and 42 were assigned to the Zygomycetes (35.6%) (Fig. 4; Fig. 5).

### Comparison of data obtained from culture-dependent and -independent investigations

**Figure 6:**
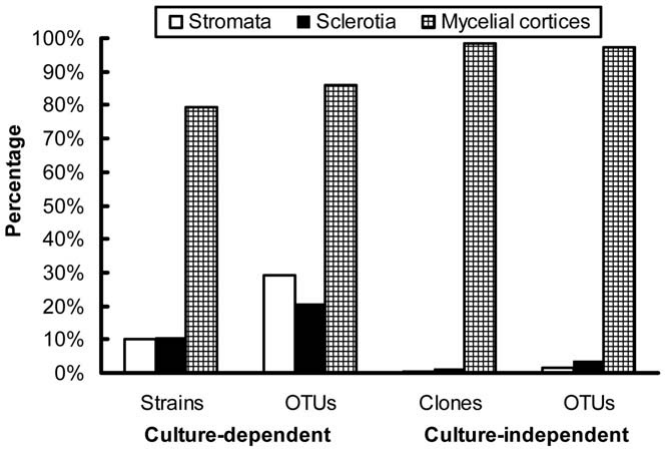
Percentage of strains/clones and OTUs detected by the culture-dependent and -independent investigations from different sections of natural *O. sinensis* specimens.

For the three different sections of natural *O. sinensis* specimens, most isolates/clones and OTUs were recovered from mycelial cortices in the two investigations. The culture-dependent method detected more OTUs originating from stromata and sclerotia than the culture-independent method (Fig. 6). The most common OTUs recovered by either culture-dependent or -independent approach belonged to the Ascomycota, followed by the Zygomycota and the Basidiomycota (Fig. 4); no members of the Chitridiomycota and the Glomeromycota were detected. The most dominant strains/clones resulting from culture-dependent and -independent study, however, were Ascomycetes and Zygomycetes, respectively (Fig. 4). Comparison of all OTUs detected by the culture-dependent and -independent investigations indicated that only 13 OTUs were in common (Table S2); the remaining OTUs were detected by only one of the two approaches, and the similarity coefficient (*Cs*) of the two investigations was only 0.124. The relative abundance of the most dominant OTU for the culture-dependent and -independent investigations was as high as 23% (132 within 572 strains) and 27% (133 within 490 clones), respectively, but they were not the same OTUs (*Pseudogymnoascus roseus* for the culture-dependent investigation and *Mucor hiemalis* for the culture-independent investigation). For the 13 shared OTUs, nine belonged to the first 20 most abundant OTUs in the culture-dependent investigation, and three belonged to the first 20 most abundant OTUs in culture-independent investigation (Table S2). Compared with the culture-dependent investigation, the culture-independent investigation detected many OTUs that had low similarities with GenBank records and that are putatively novel fungal species (Fig. 3). Data from both investigations exhibited similar rank-abundance curves (Fig. 2); these curves indicated that a few species were abundant but most were rare. A high percentage of singletons were detected by culture-dependent (42%) and culture-independent (59%) assessments (Table S1). Although the species richness index (*M*a) was a little higher with the culture-independent investigation than with the culture-dependent investigation, the two investigations generated similar diversity indices (*H'*) and evenness values (*J*) (Table S1).

## Discussion

In addition to SSCP, some other techniques have also been used for fungal community study. DGGE (denaturing gradient gel electrophoresis) and TGGE (temperature gradient gel electrophoresis) both require the use of one large PCR primer (approximately 60-mers) with regions of high GC content (GC clamps) to prevent complete strand separation during electrophoresis [44]. Such large primers may cause artifacts, which can limit the use of these techniques in community analyses [24]. Because no GC clamp primers, gradient gels, or specific apparatus are required, SSCP is simpler and more straightforward than DGGE or TGGE. Compared to RFLP (restriction fragment length polymorphism), SSCP is more sensitive; each visible band in RFLP may contain different OTUs, and RFLP patterns depend on the efficiency of endonuclease digestion [45]. Though high-throughput sequencing equipment (e.g., 454 sequencing) has been developed recently [46], the equipment is still unavailable in common laboratories, and the technique is still costly. Therefore, in this study, we use SSCP not only because it is sensitive but also because it is not expensive.

In this study, numerous fungal strains/clones from natural *O. sinensis* specimens were prescreened based on nrDNA ITS1 sequence variations through PCR-SSCP analyses. After prescreening with PCR-SSCP, sequencing was required for only 40–50% of all strains/clones, and this reduced the cost of sequencing by more than half. With the capacity to analyze 90–100 samples per day, this strategy can identify putative OTUs from numerous samples within a short time, and putative novel fungal species can be determined more easily than with other methods. In the current study, this strategy was sensitive enough to detect a fungal population that represented less than 0.2% of a fungal community.

Neither a culture-dependent nor a culture-independent method alone can describe the whole structure of a given community. Because of the intrinsic selectivity of each method, the probability of a member being detected often differs with the method. In this research, a moderate number of isolates or clones from natural *O. sinensis* specimens were studied, and many OTUs were detected in the culture-dependent and -independent investigations. Only a few of the same OTUs, however, were detected by both investigations. In comparison with the culture-independent method, the culture-dependent method detected many OTUs from stromata and sclerotia; this may be due to the enrichment of the culture-dependent method. Most OTUs detected in the culture-dependent investigation were known OTUs, while most OTUs detected in the culture-independent investigation were unknown OTUs (Fig. 3); this is reasonable, because organisms that can be cultured are more likely to have been studied in the past. More Basidiomycetes and Zygomycetes were detected in the culture-independent than in the culture-dependent investigation (Fig. 4). Low similarities between data obtained with different methods were also reported in many other cases [47], [48]. Therefore, a variety of methods should be used to study fungal community structure.

An important limitation in the current study is that different specimens were used for the culture-dependent and culture-independent investigations. It is therefore possible that at least some of the differences in the data obtained with the different investigations resulted from differences between the specimens rather than differences in the methods. However, our ongoing research with 10 specimens from different localities for both investigations supports our current results; that is, there is only few OTUs being in common between culture-dependent and -independent investigations.

Although “natural *O. sinensis* specimens” have significant pharmaceutical effects and have been used in traditional Chinese medicine for over 300 years, the commercial cultivation of this fungus on moth larvae to produce fruiting body has not been successful so far. Fungi other than *O. sinensis* originating from natural *O. sinensis* specimens could be one kind of important resources for developing alternative products of natural *O. sinensis* specimens [49], and they may also be involved in or contribute to the growth and cultivation as well as the pharmaceutical effect of natural *O. sinensis* specimens. This is the first systematic study of the fungal community in natural *O. sinensis* specimens, and large numbers of fungal species or sequences including putatively novel ones were detected.

## Supporting Information

Figure S1 **Natural** ***O. sinensis*** **specimens.** (A) The stroma and sclerotium sections. (B, C) The complex of mycelial cortices and attached soil particles outside the sclerotium as indicated by arrows.
(TIF)

Figure S2 **Representative SSCP profiles of fungal isolates (A) and clones (B, C).** For this figure, isolates were obtained from external mycelial cortices (A), and clones were obtained from stromata (B) and external mycelial cortices (C) of natural *O. sinensis* specimens. M indicated lanes of DNA markers; a nondenatured double-stranded DNA (dsDNA) ladder of 2 000, 1 000, 750, 500, and 250 bp was used as the marker. Other lanes were amplicons of nrDNA ITS1 regions from cultures (A) or clones (B, C).
(TIF)

Table S1 **Estimates of diversity indices from the culture-dependent and -independent investigations.** * Singletons are those OTUs that occur only once within the total data set of fungal cultures or clones.
(XLS)

Table S2 **Thirteen OTUs shared by the culture-dependent and -independent investigations.** ^a^ One representative strain (from the culture-dependent investigation) and clone (from the culture-independent investigation) is presented for the 13 shared OTUs. ^b^ Abundance indicates the number of strains or clones for each OTU in the entire data set. Nine OTUs detected in the culture-dependent investigation (with abundance > 6) and three OTUs detected in the culture-independent investigation (with abundance > 4) were among the 20 most dominant OTUs in the culture-dependent and -independent investigations, respectively. ^c^ BLASTN score. ^d^ Accession number of the closest database match.
(XLS)
