# Supplementary figures and images for: High Diversity of the Fungal Community Structure in Naturally-Occurring Ophiocordyceps sinensis
Source: PLoS One. 2010 Dec 15;5(12):e15570. doi: 10.1371/journal.pone.0015570 (PMC3002287; doi:10.1371/journal.pone.0015570)

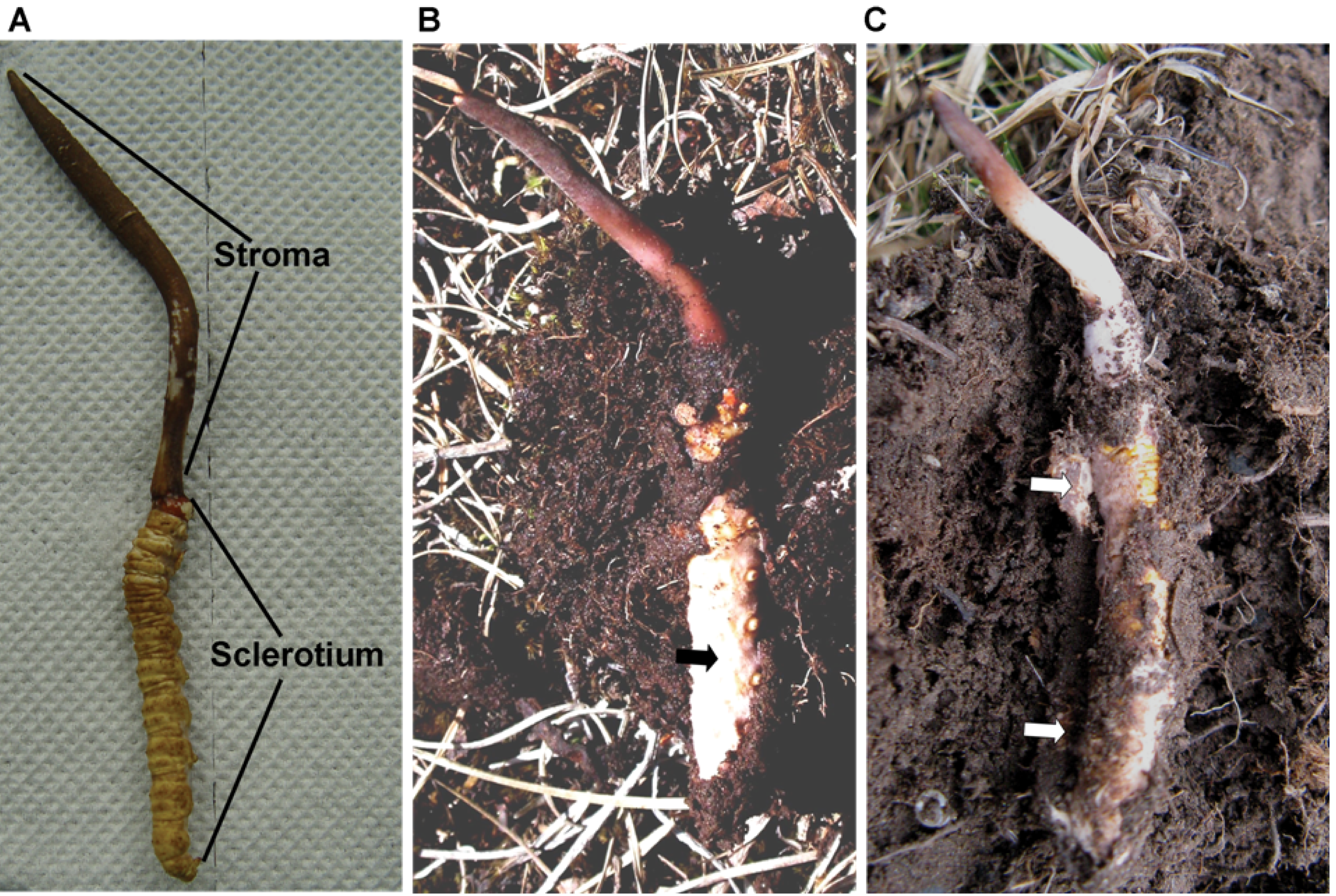

Supplement: Figure S1 — Natural O. sinensis specimens. (A) The stroma and sclerotium sections. (B, C) The complex of mycelial cortices and attached soil particles outside the sclerotium as indicated by arrows. (TIF) [file pone.0015570.s001.tif]

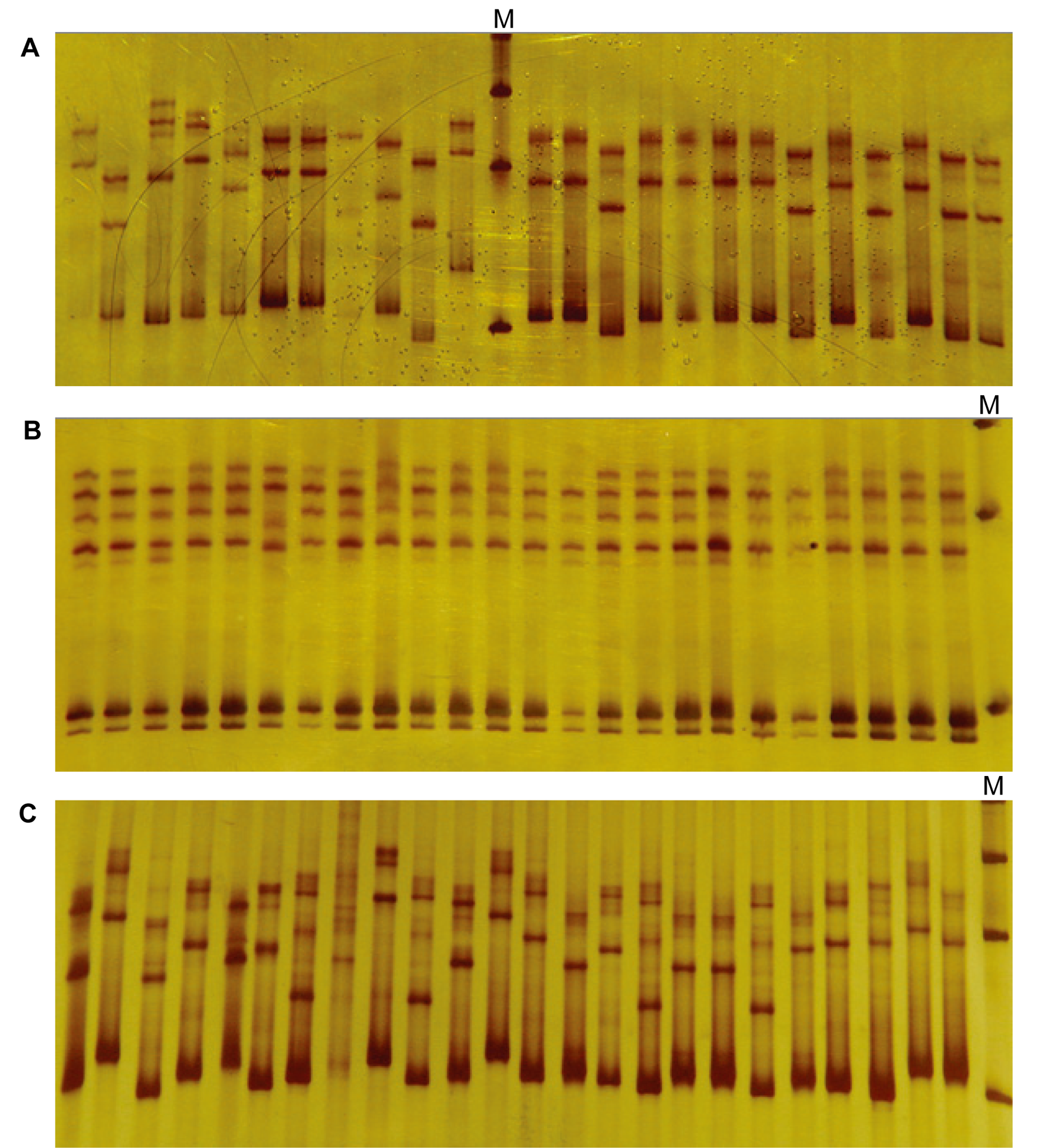

Supplement: Figure S2 — Representative SSCP profiles of fungal isolates (A) and clones (B, C). For this figure, isolates were obtained from external mycelial cortices (A), and clones were obtained from stromata (B) and external mycelial cortices (C) of natural O. sinensis specimens. M indicated lanes of DNA markers; a nondenatured double-stranded DNA (dsDNA) ladder of 2 000, 1 000, 750, 500, and 250 bp was used as the marker. Other lanes were amplicons of nrDNA ITS1 regions from cultures (A) or clones (B, C). (TIF) [file pone.0015570.s002.tif]
